# The current status and future of ADC therapy for small cell Lung Cancer: a promising approach

**DOI:** 10.1186/s12967-023-04471-2

**Published:** 2023-11-13

**Authors:** Jiawei Zhou, Peiwen Ma, Qiyu Tang, Shuhang Wang, Ning Li

**Affiliations:** 1 Clinical Cancer Center, National Clinical Research Center for Cancer/Cancer Hospital, National Cancer Center, Chinese Academy of Medical Sciences and Peking Union Medical College, Beijing, China; 2 Cancer Hospital, State Key Laboratory of Molecular Oncology, Department of Clinical Trial Center, National Cancer Center, Chinese Academy of Medical Sciences & Peking Union Medical College, Chaoyang, Beijing, China

## To the editor

Antibody-drug conjugates (ADCs) are a promising targeted therapy for cancer treatment, combining the specificity of monoclonal antibodies with the cytotoxic potency of small-molecule drugs. ADCs in small cell lung cancer (SCLC) were initially used to target DLL3, a Notch inhibitory ligand [1]. The first clinical trial showed promising objective response rates (ORR). Although, subsequent ADCs targeting DLL3 did not replicate the same clinical benefits, sparked a surge of interest in the development of ADCs [2]. In order to provide a concise summary of the current research status in this field, we have analyzed data from preclinical studies and clinical trials conducted since 2020.

## Findings of clinical trials since 2020

In our analysis of ongoing clinical trials, as presented in Table 1, we identified four trials that reported relevant findings for SCLC. Notably, the most encouraging outcomes were observed with HS-20,093 and ABBV-011. As of March 10, 2023, a total of 53 participants, including SCLC patients and individuals with other solid tumors, were enrolled in these trials. Among the subset of 9 patients, a remarkable response rate of 77.8% was observed, with 7 patients showing partial responses (PRs). These findings suggest that HS-20,093 demonstrated promising anti-tumor activity in SCLC patients [3]. Seizure-related homolog protein 6 (SEZ6) is another promising target that has demonstrated efficacy. It is a surface-expressed target with broad expression in SCLC cells and minimal expression in normal tissues, as confirmed by RNA-seq and immunohistochemistry (IHC) analysis. Subsequently, a first-in-human study of ABBV-011 in patients with SCLC was conducted [4]. In this trial, the confirmed objective response rate was 25%, with 10 patients showing PR. The median duration of response was 4.2 months. The median progression-free survival was 3.5 months.

**Table 1:** Summary of ADC trials targeting SCLC since 2020

Phase	ID	Target	Payload	Trial results	Status
I	OX-002	lymphocyte antigen 75	Microtubule inhibitor	NA	Open
I	Patritumab deruxtecan	ERBB3	Microtubule inhibitor	NA	Planned
I	BAT-8008	TROP2	DNA topoisomerase I inhibitor	NA	Open
I	ABBV-706	Seizure related 6 homologue	DNA topoisomerase I inhibitor	10 PR of 40 pts	Open
I	OMTX-705	fibroblast activation protein alpha	Microtubule inhibitor	NA	Open
I	BL-B01D1	EGFR-HER3	DNA topoisomerase I inhibitor	1 PR of 11pts	Closed
I	HS-20,093	B7-H3	DNA topoisomerase I inhibitor	7 PR of 9 pts	Open
I	FDA-018	TROP2	Unknown	NA	Open
I	DAC-002	TROP2	Microtubule inhibitor	NA	Open
I	RC-88	Mesothelin	Microtubule inhibitor	NA	Open
I/II	MHB-088 C	B7-H3	DNA topoisomerase I inhibitor	NA	Open
I/II	MHB-036 C	TROP2	DNA topoisomerase I inhibitor	NA	Open
I/II	Sacituzumab govitecan plus berzosertib	TROP2	DNA topoisomerase I inhibitor	1 PR only	Open
I/II	ESG-401	TROP2	DNA topoisomerase I inhibitor	NA^a^	Open
I/II	ARX-517	folate hydrolase 1	Microtubule inhibitor	NA^a^	Open
I/II	SKB-264	TROP2	DNA topoisomerase I inhibitor	NA^a^	Open
II	BL-B01D1	EGFR-HER3	DNA topoisomerase I inhibitor	NA	Planned
II	Ifinatamab deruxtecan	B7-H3	DNA topoisomerase I inhibitor	NA	Closed
II	Trastuzumab deruxtecan	ERBB2	Microtubule inhibitor	NA	Open

^a^Mid-term results available with no disclosure of SCLC-related findings

In addition, a few trials have reported results related to SCLC, primarily with single-case PR reports. For example, in the case of BL-B01D1, an EGFRxHER3 bispecific ADC utilizing a DNA topoisomerase I inhibitor as the payload, out of 7 SCLC patients, 1 patient showed a complete partial response (cPR) [5]. In terms of combination therapy, a phase I trial was conducted utilizing a combination of Sacituzumab Govitecan and Berzosertib, an ATR inhibitor, in SCLC patients. Tumor regressions were observed in two patients, including one patient with SCLC that had transformed from EGFR-mutant non-small cell lung cancer [6].

### Preclinical findings of SCLC targeted ADC

Pre-clinical studies suggested some potential yet unexplored targets, such as c-Kit, Glypican 2, JAM3, etc. Overexpression of c-Kit is observed in 70% of SCLC patients. In this particular investigation, researchers assessed the efficacy of an ADC specifically designed to target c-Kit with DM1 as payload. The results demonstrated that 4C9-DM1 effectively induced apoptosis in SCLC cells [7]. In a recent study, DNA-damaging dimers were used to specifically target Glypican 2 [8]. The dimers induced DNA damage, apoptosis, and bystander cell killing, resulting in regression of SCLC tumors. Higher expression levels of junctional adhesion molecule 3, known as JAM3, has been also validated in SCLC cell lines. JAM3 was detected at both mRNA and protein levels in all three SCLC cell lines. Although an ADC targeting JAM3 has not been developed yet, these findings suggest that JAM3 could be a promising therapeutic target for SCLC [9].

## Summary

The promising results of HS-20,093 and ABBV-011 demonstrate the feasibility of ADC as a therapeutic strategy. Overall, ADCs targeting SCLC are mostly in the early stages of development, including phase I or phase I/II trials. TROP2 is the most commonly targeted molecule in SCLC (7 out of 19), followed by B7-H3 (3 out of 19). Other targets are scattered. The diversity of targets is good, with a total of 10 different targets, including one advanced bi-specific ADCs in the field. In terms of payload, microtubule inhibitors and TOP1 inhibitors are the two main categories, but TOP1 inhibitors have shown efficacy in SCLC. Further research and development of ADCs could provide new treatment options for SCLC patients and improve clinical outcomes. In the development strategy for ADCs in SCLC, several aspects can be considered:

1. Target selection: It is important to avoid blindly following trends and instead consider solid preclinical data as a backup for subsequent clinical. Additionally, there are several ADCs in preclinical stages or targeting potent targets that show promising clinical effects, and their potential should be explored.
2. Payload selection: Although, microtubule inhibitors and TOP1 inhibitors are the main choices, considering the physiological characteristics of SCLC, the development of targeted drugs addressing resistance mechanisms could be an important way to achieve differentiation.
3. Beyond expression: Recent studies in related tumor types (non-small cell lung cancer, NSCLC) have reported the impact HER2 amplification and mutation on receptor internalization [10]. It is worth investigating similar mechanisms exist for current targets in SCLC as well. Expression may no longer be the criterion for ADC application.

In conclusion, ADCs offer a promising and rapidly evolving therapeutic strategy for the treatment of SCLC. To maximize their potential, a comprehensive approach is needed, including careful target selection, optimization of payloads to overcome drug resistance, and exploration of mechanisms to enhance ADC efficiency. Continued research and development efforts in these areas are expected to further improve the efficacy and safety of ADCs, ultimately benefiting patients with SCLC in clinical practice.

## Data Availability

Not Applicable.

## Abbreviations

ADCs: Antibody-drug conjugates
SCLC: Small cell lung cancer
ORR: Objective response rates
PRs: Partial responses
SEZ6: Seizure-related homolog protein 6
IHC: Immunohistochemistry
cPR: Complete partial response
NSCLC: Non-small cell lung cancer
